# Human coronavirus OC43 nanobody neutralizes virus and protects mice from infection

**DOI:** 10.1128/jvi.00531-24

**Published:** 2024-05-06

**Authors:** Amy Adair, Li Lynn Tan, Jackson Feng, Jason Girkin, Nathan Bryant, Mingyang Wang, Francesca Mordant, Li-Jin Chan, Nathan W. Bartlett, Kanta Subbarao, Phillip Pymm, Wai-Hong Tham

**Affiliations:** 1 The Walter and Eliza Hall Institute of Medical Research, Parkville, Victoria, Australia; 2 Department of Medical Biology, The University of Melbourne, Melbourne, Victoria, Australia; ^3^College of Health, Medicine and Wellbeing, University of Newcastle, Callaghan, New South Wales, Australia; 4 Infection Research Program, Hunter Medical Research Institute, New Lambton Heights, New South Wales, Australia; 5 Department of Microbiology and Immunology, The Peter Doherty Institute for Infection and Immunity, University of Melbourne, Melbourne, Victoria, Australia; 6 WHO Collaborating Centre for Reference and Research on Influenza, The Peter Doherty Institute for Infection and Immunity, University of Melbourne, Melbourne, Victoria, Australia; Loyola University Chicago - Health Sciences Campus, Maywood, Illinois, USA

**Keywords:** X-ray crystallography, nanobodies, anti-viral therapeutics, coronavirus

## Abstract

Human coronavirus (hCoV) OC43 is endemic to global populations and usually causes asymptomatic or mild upper respiratory tract illness. Here, we demonstrate the neutralization efficacy of isolated nanobodies from alpacas immunized with the S1_B_ and S1_C_ domain of the hCoV-OC43 spike glycoprotein. A total of 40 nanobodies bound to recombinant OC43 protein with affinities ranging from 1 to 149 nM. Two nanobodies WNb 293 and WNb 294 neutralized virus at 0.21 and 1.79 nM, respectively. Intranasal and intraperitoneal delivery of WNb 293 fused to an Fc domain significantly reduced nasal viral load in a mouse model of hCoV-OC43 infection. Using X-ray crystallography, we observed that WNb 293 bound to an epitope on the OC43 S1B domain, distal from the sialoglycan-binding site involved in host cell entry. This result suggests that neutralization mechanism of this nanobody does not involve disruption of glycan binding. Our work provides characterization of nanobodies against hCoV-OC43 that blocks virus entry and reduces viral loads *in vivo* and may contribute to future nanobody-based therapies for hCoV-OC43 infections.

**IMPORTANCE:**

The pandemic potential presented by coronaviruses has been demonstrated by the ongoing COVID-19 pandemic and previous epidemics caused by severe acute respiratory syndrome coronavirus and Middle East respiratory syndrome coronavirus. Outside of these major pathogenic coronaviruses, there are four endemic coronaviruses that infect humans: hCoV-OC43, hCoV-229E, hCoV-HKU1, and hCoV-NL63. We identified a collection of nanobodies against human coronavirus OC43 (hCoV-OC43) and found that two high-affinity nanobodies potently neutralized hCoV-OC43 at low nanomolar concentrations. Prophylactic administration of one neutralizing nanobody reduced viral loads in mice infected with hCoV-OC43, showing the potential for nanobody-based therapies for hCoV-OC43 infections.

## INTRODUCTION

Coronaviruses are enveloped, positive-stranded RNA viruses which infect a wide range of mammalian species. The zoonotic threat and pandemic potential presented by coronaviruses have been demonstrated by the ongoing COVID-19 pandemic caused by severe acute respiratory syndrome coronavirus 2 (SARS-CoV-2), resulting in over 600 million infections and over 6 million deaths worldwide. Within the last two decades, two epidemics have also been caused by SARS-CoV and Middle East respiratory syndrome coronavirus (MERS-CoV), which led to approximately 600 and 800 deaths, respectively (1, 2).

Outside of these major pathogenic coronaviruses, there are four endemic coronaviruses that infect humans: hCoV-OC43, hCoV-229E, hCoV-HKU1, and hCoV-NL63. Together, these coronaviruses are estimated to cause approximately 15%–30% of common colds in humans, with hCoV-OC43 shown to account for the highest proportion (3). hCoV-OC43 was first isolated in 1967 from patients who presented with upper respiratory disease, though molecular clock analysis has pointed toward its original emergence in humans from a rodent reservoir via a livestock species in the 1890s (4–6). Generally, hCoV-OC43 presents with mild upper-respiratory tract symptoms; however, the virus has been shown to have neuroinvasive properties as well as being capable of causing more severe disease and fatal pneumonia in rare cases in children, the elderly, and the immunocompromised (7–9).

On the virion surface, hCoV-OC43 expresses a trimeric spike protein which is critical for viral entry, host range, and tissue tropism and is a major target of the host immune response (10). Spike proteins undergo major conformational changes in their transition from prefusion to post-fusion states. Each monomer consists of two major functional subunits—S1 and S2. The S1 subunit is further divided into four distinct domains (S1_A–D_) (Fig. S1A). The S1 subunit is responsible for host receptor recognition, whereas the S2 subunit is involved in membrane fusion. Structural characterization shows how OC43 S1_A_ (also known as the N-terminal domain) mediates viral attachment to host 9-*O*-acetylated sialic acids (11). Interestingly, the S1_B_ domain (also known as the C-terminal domain), which among other human coronaviruses is typically responsible for binding to protein receptors, has no such role yet elucidated for hCoV-OC43.

While the human infective betacoronaviruses share a conserved fold in the S1_B_ core, the end distal from spike in the open conformation is largely structurally unique to each hCoV, with the exception of similar distal end structures in SARS-CoV-1 and SARS-CoV-2 (12, 13). This distal section contains the receptor-binding motif (RBM) in all other human infective betacoronaviruses that directly engages with the host receptor for cell entry (12–17). Despite a relative lack of sequence information compared to SARS-CoV-2, and epidemic hCoVs SARS-CoV-1 and MERS-CoV, there is considerable variation in the OC43 S1_B_ domain. This is particularly the case in regions which overlap the receptor-binding motifs in other hCoVs (Fig. S1B and C).

Monoclonal antibodies (mAbs) are important anti-viral treatments in the global effort against the COVID-19 pandemic (18). Given the essential role of S1_B_ in receptor binding for viral entry, it is the major antigenic site recognized by neutralizing mAbs across infective human coronaviruses. Over 5,000 mAbs have been described against SARS-CoV-2 S1_B_ (19), including ones with therapeutic potential (20, 21). S1_B_- binding neutralizing antibodies have also been described for all human endemic coronaviruses (22–32). Although a collection of mAbs against hCoV-OC43 has been described, none are currently in clinical use (33–40). Anti-hCoV-OC43 mAbs have been generated by either immunizing mice with hCoV-OC43 (33), MERS-CoV (34), or SARS-CoV-2 spike (35, 36), or derived from SARS-CoV-2 convalescent patients (37–39). SARS-CoV-2- and MERS-CoV-derived mAbs, which showed cross-recognition of hCoV-OC43, bind to conserved epitopes on the S2 subunit, specifically the stem helix region (34–39). However, the majority of these cross-reactive mAbs have little to no neutralizing activity against hCoV-OC43 S vesicular stomatitis virus pseudotypes (35). Recently, over 22 chimeric neutralizing mAbs obtained from mouse immunization were mapped to the S1 subunit of hCoV-OC43 (33). Four neutralizing S1_A_ mAbs compete with sialic acid for binding to the OC43 spike. Using cryo-EM, S1_A_-targeting mAb 46C12 was confirmed to bind to the sialoglycan-binding site and represents one of the more potent neutralizing antibodies against hCoV-OC43. Cryo-EM also revealed two unique epitopes on the S1_B_ subunit where 43E6 bound close to the threefold symmetry axis and 37F1 bound distal to the threefold symmetry axis. A further three mAbs were found to bind to the S1_B_, but cryo-EM only showed the apo-form of the prefusion spike trimer. It is speculated that the binding sites were inaccessible in the prefusion spike conformation and required opening of the S1_B_ domain. Thus, hydrogen-deuterium exchange mass spectrometry and single-substitution mutagenesis were performed, revealing residues 538 and 404 as crucial for binding of the neutralizing mAb. Collectively, binding of S1_B_-directed mAbs did not compete in sialoglycan-binding assays and are thought to neutralize infection through an alternative mechanism (33). None of these neutralizing antibodies have been tested in an *in vivo* model of hCoV-OC43 infection. Nanobodies contain the smallest natural antigen-binding fragment consisting of only a heavy chain domain and are present in camelids (41). They maintain high-affinity recognition of antigen while having improved stability across pH and temperature ranges compared with conventional mAbs. Multiple nanobodies have been identified as potential therapeutics against SARS-CoV-2 and other human coronaviruses, and their small size allows a wide variety of multivalent display formats to increase potency (42–48). Their increased stability also allows for alternative delivery mechanisms to be explored, including inhaled delivery for respiratory diseases (49, 50). To the best of our knowledge, no nanobodies have been characterized against hCoV-OC43. Here, we identified nanobodies by screening a phage display library generated from an alpaca immunized with hCoV-OC43 S1_B_ and S1_C_ domains. Two high-affinity nanobodies showed potent neutralization against hCoV-OC43 and reduced viral loads in a mouse model of respiratory infection. In addition, we provide structural characterization of a neutralizing nanobody against hCoV-OC43.

## RESULTS

### High-affinity hCoV-OC43 RBD-binding nanobodies that neutralize virus

**Fig 1:**
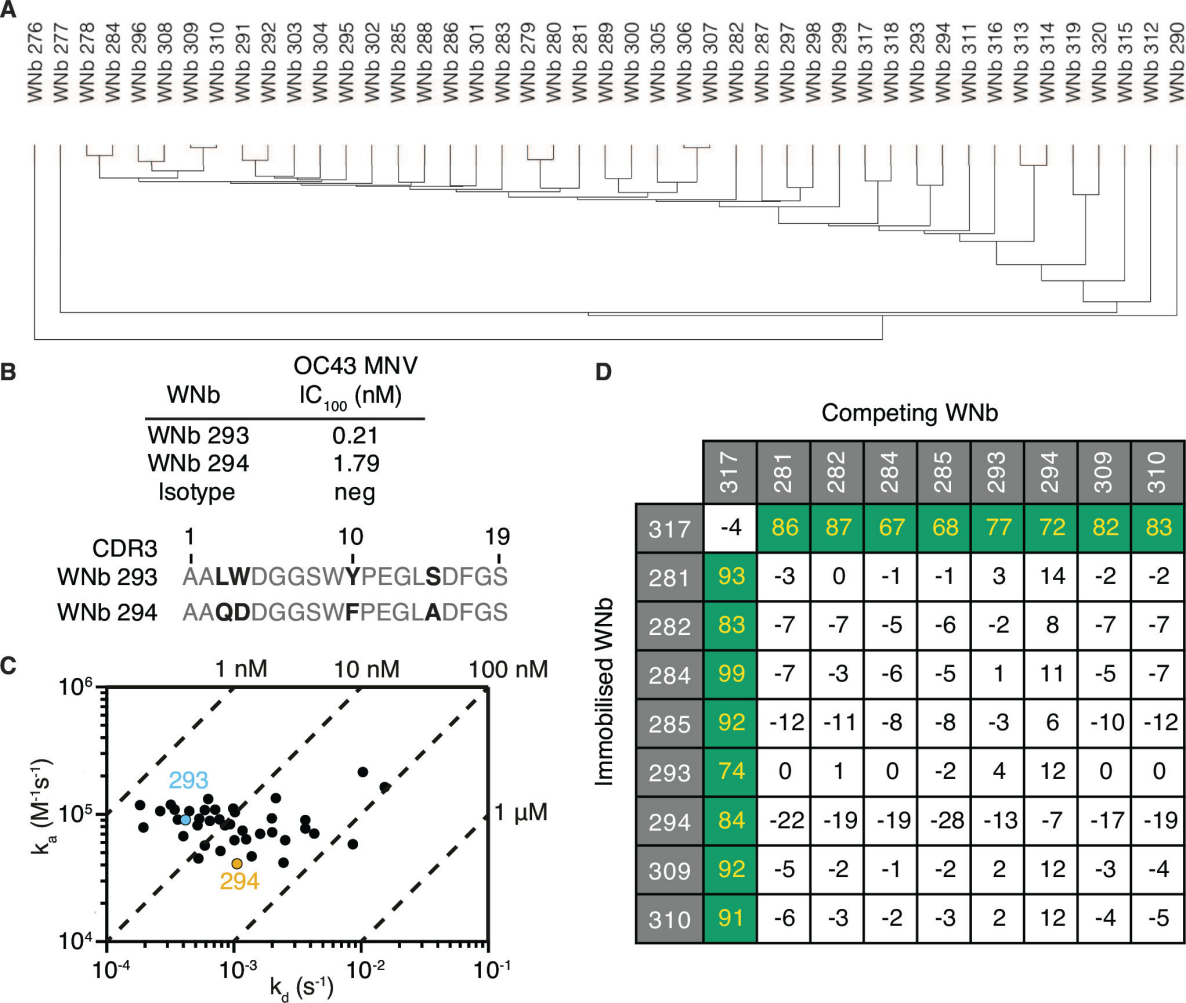
Identification and functional characterization of neutralizing anti-OC43 nanobodies. (**A**) Nanobodies are ordered based on their clonal lineage as determined by their CDR3 sequences. (**B**) MNV IC_100_ values shown are the geometric mean of *n* = 2–4 biological replicates (top). Alignment of CDR3 WNb 293 and 294 nanobodies with amino acid differences highlighted in black (bottom). (**C**) Iso-affinity plot showing the dissociation rate constants (*k*_*d*_) and association rate constants (*k*_*a*_) of WNb nanobodies as measured by bio-layer interferometry. Symbols that fall on the same diagonal lines have the same equilibrium dissociation rate constants (*K*_*D*_) indicated on the top and right sides of the plot. WNb 293 and 294 are highlighted in blue and orange, respectively. (**D**) Epitope competition experiments using bio-layer interferometry using immobilized nanobodies indicated on the left column incubated with nanobodies indicated on the top row pre-incubated with OC43 S1_B+C_ using a 10:1 molar ratio. Binding of RBD premixed with nanobody was calculated relative to RBD binding alone, which was assigned to 100%. The green and white boxes represent non-competing and competing nanobodies, respectively.

To identify nanobodies that are effective at neutralizing hCoV-OC43, we immunized an alpaca with recombinant hCoV-OC43 protein which encompassed the S1_B_ and S1_C_ domains (OC43 S1_B+C_) (Fig. S1A). We generated a nanobody phage display library from the alpaca and performed two rounds of phage display panning. Sequence alignment and phylogenetic analyses of positive phage supernatants from ELISA-based screening identified 45 distinct nanobody clonal groups based on at least one amino acid difference in the complementary determining region 3 (CDR3), which we named WNb 276 to WNb 320 (Fig. 1A; Table 1). The CDR3 lengths varied between 11 and 19 residues. The 45 nanobodies were expressed and purified with yields ranging from 0.7 to 2.9 mg (Fig. S2; Table 2). We tested the potential of the 45 nanobodies to neutralize hCoV-OC43 virus using a microneutralization assay (MNV) with viral cytopathic effect as a measurement of infectivity (51). Two nanobodies WNb 293 and WNb 294 neutralized virus at 0.21 and 1.79 nM, respectively (Fig. 1B; Table 2). Compared to the other 43 nanobodies, the CDR3s of WNb 293 and WNb 294 are more like each other, with four amino acid differences in their CDR3 between them (Fig. 1B; Table 1).

**TABLE 1:** Nanobody sequences generated against OC43 S1_B+C_^*a*^

	Antibody sequence	Antibody sequence	Antibody sequence	Antibody sequence
	CDR1	CDR2	CDR3	
	Heavy chain	Heavy chain	Heavy chain	VHH
WNb 276	GFTFSNYG	IDSDGTIT	IKVMDWVPGRAPPF	QVQLQESGGGLVQSGGSLRLSCAASGFTFSNYGMMWVRQAPGKGVEWVSRIDSDGTITLYKDSVKGRFTISRDNAKNMLYLQMESLKPEDTALYYCIKVMDWVPGRAPPFRGQGTQVTVSS
WNb 277	GSTSSITA	AITNDGRT	RTWRDFGPGS	QVQLQESGGGSVQPGQSLRLSCAASGSTSSITAMGWHRRQAPGQDRERVAAITNDGRTTYADAVKGRFTISRDNAKNTVYLQMDSLKPEDTAVYYCRTWRDFGPGSWGQGTQVTVSS
WNb 278	GRTFSDYF	ISWSGGTT	ATAIDYGLGLTVEYEYDY	QVQLQESGGGLVQAGGSLRLSCAASGRTFSDYFMGWFRQAPGMEREFVASISWSGGTTYYADSVKGRFTISRDNTKNTVYLQMNSLKPEDTAVYYCATAIDYGLGLTVEYEYDYWGQGTQVTVSS
WNb 279	GRTFSDYF	ITWSGGST	AGGLDMGWSRVALGEYDY	QVQLQESGGGLVQPGGSLRLSCAASGRTFSDYFMGWFRQAPGKEREFVAAITWSGGSTYYTDSVKGRFTISRDNAKNTVYLQMNSLKPEDTAVYYCAGGLDMGWSRVALGEYDYWGQGTQVTVSS
WNb 280	GRTFSNYF	ISWSGGST	AGGLDMGWSRVNLGEYDY	QVQLQESGGGLVQAGGSLRLSCAASGRTFSNYFMGWFRQAPGKEREFVAAISWSGGSTYYTDSVKGRFTISRDNAKNTVYLQMNSLKPEDTAVYYCAGGLDMGWSRVNLGEYDYWGQGTQVTVSS
WNb 281	GRTFSDYF	ISWSGGST	AAALDEGWARVAGPEYDY	QVQLQESGGGLVQAGGSLRLSCAASGRTFSDYFMGWFRQAPGKEREFVTAISWSGGSTYYADSVKGRFTISRDNAKNTVFLQMNSLKPEDTAVYYCAAALDEGWARVAGPEYDYWGQGTQVTVSS
WNb 282	GRTFSDYF	ISWSGGTT	AAARDMGWGRTGENEYDY	QVQLQESGGGLVQAGDSLRLSCAASGRTFSDYFMGWFRQAPGKEREFVASISWSGGTTYYADSVKGRFTISRDNAKNTVYLQMNRVKSEDTAVYYCAAARDMGWGRTGENEYDYWGQGTQVTVSS
WNb 283	GRSFSDYF	ISWSGGST	AADWDYGWARTGTDEYDY	QVQLQESGGGLVQAGGSLRLSCAASGRSFSDYFMGWFRQAPGKEREFVASISWSGGSTYYADSVKGRFTISRDNAKNTVYLQMNSLKPENTAVYYCAADWDYGWARTGTDEYDYWGQGTQVTVSS
WNb 284	GRTFSDYF	ISWSGGTT	AAAIDYGLGLTVEYEYDY	QVQLQESGGGLVQAGGPLRLSCAASGRTFSDYFMGWFRQAPGMEREFVASISWSGGTTYYADSVKGRFTISRDNAKNTVYLQMNSLKPEDTAVYYCAAAIDYGLGLTVEYEYDYWGQGTQVTVSS
WNb 285	GRTFSDYA	ISWSGGTT	AADYDYGWGRVRVSEYDY	QVQLQESGGGLVQAGGSLRLSCAASGRTFSDYAMGWFRQAPGKEREFVAAISWSGGTTYYADSVKGRFTISRDNAKNTVYLQMNSLTPEDTAVYYCAADYDYGWGRVRVSEYDYWGQGTQVTVSS
WNb 286	GRTFSSYA	ISWSGGTT	AAAVDYGLGLYRVGEYDS	QVQLQESGGGLVQAGGSLRLSCAASGRTFSSYAMGWFHQAPGKEREFVASISWSGGTTYYADSVKGRFTISRDNAKNTVYLQMNSLKPEDTAVYYCAAAVDYGLGLYRVGEYDSWGQGTQVTVSS
WNb 287	TFSFSDYA	ISWSGGTT	AAEIDRGLLLHAVYEYDY	QLQESGGGLVQPGGSLRLSCAASGRTFSFSDYAMGWFRQAPGKEREFVASISWSGGTTYYADSVKGRFTISRDNAKNTVYLQMNSLKPEDTAVYYCAAEIDRGLLLHAVYEYDYWGQGTQVTVSS
WNb 288	GRTFSDYF	ISWSGGST	AADHDRGWARLRLAEYDY	QVQLQESGGGLVQAGGSLRLSCAASGRTFSDYFMGWFRQAPGKEREFVAAISWSGGSTYYADSVKGRSTISRDNAKNTVYLQMNSLKPEDTAVYYCAADHDRGWARLRLAEYDYWGQGTQVTVSS
WNb 289	GRTFSDYA	ISWSGGTT	AAVMDLGMLAADGYEYDY	QVQLQESGGGLVQAGGSLRLSCAASGRTFSDYAMGWFRQAPGKEREFVATISWSGGTTYYADSVKGRFTISRDNAKNTVYLQMNSLKPEDTAVYYCAAVMDLGMLAADGYEYDYWGQGTQVTVSS
WNb 290	TVTFNTYT	ITWNEGRP	AADERFGDSLERS	QVQLQESGGGLVQSGGSLTLSCRASTVTFNTYTMAWFRQTPDKEREFVAAITWNEGRPSYADFVKGRFTISRDNAKSTVFLQMNNLRPEDTGIYYCAADERFGDSLERSWGQGTQVTVSS
WNb 291	GRTFSNYF	ISWSGGTT	AANTDYGMGFVRAYEYDY	QVQLQESGGGLVQAGGSLRLSCAASGRTFSNYFMGWFRQAPGKEREFVAAISWSGGTTYYADSVKGRFTISRDNAKNTVYLQMNSLKPEDTAVYYCAANTDYGMGFVRAYEYDYWGQGTQVTVSS
WNb 292	GRTFSDYA	ISWSGGTT	AANTDYGLGLVRAYEYDY	QVQLQESGGGLVQAGASLRLSCAASGRTFSDYAMGWFRQAPGKEREFVAAISWSGGTTYYADSVKGRFTISRDNAKNTVYLQMNSLKPEDTAVYYCAANTDYGLGLVRAYEYDYWGQGTQVTVSS
WNb 293	GRTFSSYA	ISWSGGTT	AALWDGGSWYPEGLSDFGS	QVQLQESGGGSVQAGDSLRLSCVASGRTFSSYALGWFRRAPGKEREFVAAISWSGGTTYYADSVKGRFTISRDNAKNTVYLQMNSLKPEDTAVYYCAALWDGGSWYPEGLSDFGSWGQGTQVTVSS
WNb 294	GRTFSSYA	ISWSGGTT	AAQGDGGSWFPEGLADFGS	QVQLQESGGGLVQAGGSLRLSCAASGRTFSSYAMGWFRQAPGKEREFVAAISWSGGTTYYGDSVKGRFTISRDNAKNTVYLQMNSLKPEDTAVYYCAAQGDGGSWFPEGLADFGSWGQGTQVTVSS
WNb 295	GRTFSSYA	ISWSGGTT	AAAEDYGLGLTTSYEYDY	QVQLQESGGGLVQAGGSLRLSCAASGRTFSSYAMGWFRQAPGKEREFVGGISWSGGTTYYADSVKGRFTISRDNAKNTVYLQMNSLKPDDTAVYYCAAAEDYGLGLTTSYEYDYWGQGTQVTVSS
WNb 296	GRTFSDYF	ISWSGGTT	AAVADYGLGLTTSYEYDY	QVQLQESGGGLVQAGGSLRLSCAASGRTFSDYFMGWFRQAPGKEREFVASISWSGGTTYYADSVKGRFTISRDNANNTVYLQMNSLKPVDTAVYYCAAVADYGLGLTTSYEYDYWGQGTQVTVSS
WNb 297	GRTFSDYA	ISWSGGTT	AADTDYGMGLITVPDFGS	QVQLQESGGGSVQAGGSLRLSCAASGRTFSDYAMGWFRQAPGKEREFVASISWSGGTTYYADSVKGRFTISRDNAKNTVYLQMNSLKPEDTAVYYCAADTDYGMGLITVPDFGSWGQGTQVTVSS
WNb 298	GRTFSDYF	ISWSGGTT	AAEIDYGLGLHAAADFGS	QVQLQESGGGLVQPGGSLRLSCAASGRTFSDYFMGWFRQAPGKEREFVASISWSGGTTYYADSVKGRFTISRDNAKNTVYLQMNSLKPEDTAVYYCAAEIDYGLGLHAAADFGSWGQGTQVTVSS
WNb 299	GFTFSNYA	ISWSGGTT	AAASDYGMVQMSDYEYDY	QVQLQESGGGWVQPGGSLRLSCAASGFTFSNYAMGWFRQAPGKEREFVATISWSGGTTVYADSVKGRFTISRDNAKNTVYLQMNSLKPEDTAVYYCAAASDYGMVQMSDYEYDYWGQGTQVTVSS
WNb 300	GRTFSDYA	ISWSGGTT	AAAWDRGMVVAGPYEYDY	QVQLQESGGGLVQAGGSLRLSCAASGRTFSDYAMGWFRQAPGKEREFVATISWSGGTTYYGDSVKGRFTISRDNAKNTVYLQMNSLKPEDTAVYYCAAAWDRGMVVAGPYEYDYWGQGTQVTVSS
WNb 301	GRTFSSYA	ISWSGGTT	AAAIDHGMGLERVDEFDY	QVQLQESGGGLVQAGGSLRLSCAASGRTFSSYAMGWFRQAPGKEREFVASISWSGGTTYYSDSVKGRFTISRDNAKNTVYLQMNSLKPEDTAVYNCAAAIDHGMGLERVDEFDYWGQGTQVTVSS
WNb 302	GRTFSSYF	ISWSGGTT	AAAIDYGLGLTRVAEFDY	QVQLQESGGGLVQAGDSLRLSCAASGRTFSSYFMGWFRQAPGKEREFVATISWSGGTTYYADSVKGRFTISRDNTKNTVYLQMNSLKPEDTAVYYCAAAIDYGLGLTRVAEFDYWGQGTQVTVSS
WNb 303	GRTFSDYA	VSWSGGTT	AATEDYGMGLLADYEYDY	QVQLQESGGGLVQAGGSLRLSCAASGRTFSDYAMGWFRQAPGKEREFVSAVSWSGGTTYYADSVKGRFTIARDNAKNTVYLQMNSLKPEDTAVYYCAATEDYGMGLLADYEYDYWGQGTQVTVSS
WNb 304	ARTFSDYA	ISWSGGTT	AAEPDYGMGLTASYEYNY	QVQLQESGGGLVQAGGSLRLSCAASARTFSDYAMGWFRQAPGKEREFVASISWSGGTTYYADSVKGRFTISRDNAKNTVYLQMNSLKPEDTAVYYCAAEPDYGMGLTASYEYNYWGQGTQVTVSS
WNb 305	GRTFSDYF	ISWSGGSV	AASLDNGMALFGPIEYDY	QVQLQESGGGLVQAGGSLRLSCAASGRTFSDYFMGWFRQAPGKEREFVAAISWSGGSVYYADSVKGRFTISRDNAKKTVYLQMNSLKPEDTAVYYCAASLDNGMALFGPIEYDYWGQGTQVTVSS
WNb 306	GRTFSDYF	ISWSGGST	AAVLDRGMAAMGPEEYDY	QVQLQESGGGLVQAGGSLRLSCAASGRTFSDYFMGWFRQTPGKEREFVAAISWSGGSTYYADSVKGRFTISRDNAKNTVYLQMNRLKPEDTAVYYCAAVLDRGMAAMGPEEYDYWGQGTQVTVSS
WNb 307	GRTFSDYF	ISWSGGST	AAVLDRGMATMGPEEYDY	QVQLQESGGGLVQAGGSLRLSCAASGRTFSDYFMGWFRQTPGKEREFVAAISWSGGSTYYADSVKGRFTISRDNAKNTVYLQMNRLKPEDTAVYYCAAVLDRGMATMGPEEYDYWGQGTQVTVSS
WNb 308	GRTFSDYF	ISWSGGTT	AAVADYGLGLTDADDYDY	QVQLQESGGGLVQAGGSLRLSCAASGRTFSDYFMGWFRQAPGKEREFVASISWSGGTTYYADSVKGRFTISRDNANNTVYLQMNSLKPVDTAVYYCAAVADYGLGLTDADDYDYWGQGTQVTVSS
WNb 309	GRTFSDYF	ISWSGGTT	AAAADYGMGLTNADDYEY	QVQLQESGGGLVQAGGSLRLSCAASGRTFSDYFMGWFRQAPGKAREFAASISWSGGTTYYADSVKGRFTISRDNAKNTVYLQMNSLKPEDTAVYYCAAAADYGMGLTNADDYEYWGQGTQVTVSS
WNb 310	GRTFSDYF	ISWSGGTT	AAVADYGMGLTNADDYEY	QVQLQESGGGLVQAGGSLRLSCAASGRTFSDYFMGWFRQAPGKAREFAASISWSGGTTYYADSVKGRFTISRDNAKNTVYLQMNSLKPEDTAVYYCAAVADYGMGLTNADDYEYWGQGTQVTVSS
WNb 311	GRTFSSKA	ISWSGGST	APRTIATMTRPYEYDY	QVQLQESGGGLVQAGGSLRLSCAASGRTFSSKAMAWFRQAPGKEREFVAAISWSGGSTHYADSVEGRFTISRDNAKNTVYLQMNSLKPEDTAVYYCAPRTIATMTRPYEYDYWGQGTQVTVSS
WNb 312	GSIGSINA	ITSGGST	NARWILYNSHPLRGMDY	QVQLQESGGGLVQPGGSLRLSCVASGSIGSINAMGWYRQVPGKERELVADITSGGSTSYADSVKGRFTISRDNAKNTVYLQMNSLKPEDTAVYYCNARWILYNSHPLRGMDYWGKGTQVTVSS
WNb 313	GRTFSSYA	RGWSGGST	AAGDIVQVTTGEGSGY	QVQLQESGGGLVQAGGSLRLSCAASGRTFSSYAMGWFRQAPGKEREFVAARGWSGGSTYYADSVKGRFTISRDNAKNMVYLQMNNLRPEDAAVYYCAAGDIVQVTTGEGSGYWGQGTQVTVSS
WNb 314	GRTFSSYA	RGWSGGST	AVGDIVQVTTGEGSGY	QVQLQESGGGLVQAGGSLRLSCAASGRTFSSYAMGWFRQAPGKEREFVAARGWSGGSTYYADSVKGRFTISRDNAKNTVYLLMNSLKPEDTAVYYCAVGDIVQVTTGEGSGYWGQGTQVTVSS
WNb 315	GRSLSTYA	VSWNGGRT	AAGNFVEHADAYMY	QVQLQESGGGLVQAGGSLGLACAAPGRSLSTYAMAWFRQVAGNEREFVAAVSWNGGRTYYANSVKGRFTISRDRGKNTVYLQMSSLKPEDTAVYYCAAGNFVEHADAYMYWGQGTQVTVSS
WNb 316	GRTFSSYG	INYSGDLT	AADRTATHRDYDY	QVQLQESGGGLVQPGGSLRLSCAASGRTFSSYGMGWFRQAPGKEREFVAAINYSGDLTYYADSVKGRFTISRDNAKNTVYLQMNSLKPEDTAVYYCAADRTATHRDYDYWGQGTQVTVSS
WNb 317	VRTFSNYA	ISWSGDGP	AASYLSLNFPDDL	QVQLQESGGGLVQAGGSLRLSCAASVRTFSNYAMGWFRQAPGKEREFVAAISWSGDGPYYADSVKGRFTISRDNAKNTVYLQMNSLKPEDTAVYYCAASYLSLNFPDDLRGQGTQVTVSS
WNb 318	GRTFSSYA	ISWSGGTT	AASYLSLNFPDDY	QVQLQESGGGLVQAGGSLRLSCAASGRTFSSYAMGWFRQAPGKEREFVAAISWSGGTTYYADSVKGRFTISRDNANNTVYLQMNSLKPEDTAVYYCAASYLSLNFPDDYRGQGTQVTVSS
WNb 319	GGTFDDYA	LSSSDGST	AADYLGLCWGNDDYDY	QVQLQESGGGLVQAGGSLRLSCAASGGTFDDYAIAWFRQAPGKEREGVSCLSSSDGSTYYADSVKGRFTISSDNAKNTVYLQMNSLKPEDTAVYYCAADYLGLCWGNDDYDYWGQGTQVTVSS
WNb 320	TFRFDDYA	ISSSDGSA	ASDYVGLCWGTSDYGY	QLQESGGGLVQAGGSLRLSCAASGFTFRFDDYAIGWFRQAPGKEREGVSCISSSDGSAYYADSVKGRFTISRDNAKNTVYLQMNSLKPEDTAVYYCASDYVGLCWGTSDYGYWGQGTQVTVSS

^*a*^ Amino acid sequences are shown for OC43 S1_B+C_-binding nanobodies including the CDR1, CDR2, and CDR3 loops and full-sequence information.

**TABLE 2:** Binding affinities, neutralization values, and expression yields for OC43 S1_B+C_ nanobodies^*a*^

	Binding affinities					Neutralization		Expression
	OC43 S1_B + C_ (mean ± SD)					OC43		250 mL
	*K*_*D*_ (nM)	*k*_*a*_ (× 10^5^ M^−1^s^−1^)	*k*_*d*_ (× 10^−5^ s^−1^)	Full *X*^2^	Full *R*^2^	MNV (mg/mL)	MNV (nM)	Yield (mg)
WNb 276	6.46 (± 1.29)	0.82 (± 0.09)	51.82 (± 4.75)	0.04 (± 0.02)	0.999 (± 0.00)	neg	neg	2.3
WNb 277	NB^*b*^	NB	NB	NB	NB	neg	neg	1.3
WNb 278	5.93 (± 0.01)	0.68 (± 0.01)	40.00 (± 0.65)	0.01 (± 0.00)	1.000 (± 0.00)	neg	neg	0.8
WNb 279	149.00 (± 15.00)	0.58 (± 0.04)	860.00 (± 24.50)	0.04 (± 0.00)	0.977 (± 0.00)	neg	neg	1.4
WNb 280	22.86 (± 0.30)	0.70 (± 0.00)	160.90 (± 2.00)	0.06 (± 0.01)	0.999 (± 0.00)	neg	neg	2.2
WNb 281	1.55 (± 0.01)	1.18 (± 0.07)	18.33 (± 1.20)	0.03 (± 0.02)	1.000 (± 0.00)	neg	neg	2.0
WNb 282	2.46 (± 0.25)	0.79 (± 0.03)	19.47 (± 2.70)	0.04 (± 0.01)	1.000 (± 0.00)	neg	neg	1.9
WNb 283	NB	NB	NB	NB	NB	neg	neg	0.7
WNb 284	4.01 (± 0.37)	0.91 (± 0.03)	36.29 (± 2.20)	0.02 (± 0.01)	1.000 (± 0.00)	neg	neg	1.4
WNb 285	4.23 (± 0.10)	1.06 (± 0.06)	44.70 (± 1.50)	0.03 (± 0.00)	1.000 (± 0.00)	neg	neg	1.4
WNb 286	58.80 (± 1.75)	0.42 (± 0.02)	245.00 (± 2.50)	0.08 (± 0.02)	0.996 (± 0.00)	neg	neg	2.0
WNb 287	11.22 (± 1.00)	0.84 (± 0.07)	93.32 (± 1.25)	0.04 (± 0.01)	1.000 (± 0.00)	neg	neg	2.0
WNb 288	19.57 (± 0.15)	0.64 (± 0.01)	124.45 (± 3.50)	0.03 (± 0.01)	1.000 (± 0.00)	neg	neg	2.1
WNb 289	4.73 (± 0.77)	1.32 (± 0.04)	62.85 (± 12.05)	0.05 (± 0.02)	1.000 (± 0.00)	neg	neg	2.9
WNb 290	NB	NB	NB	NB	NB	neg	neg	0.8
WNb 291	11.70 (± 0.40)	0.45 (± 0.00)	52.70 (± 1.90)	0.02 (± 0.00)	1.000 (± 0.00)	neg	neg	1.1
WNb 292	15.61 (± 0.60)	0.75 (± 0.00)	116.40 (± 4.50)	0.07 (± 0.04)	1.000 (± 0.00)	neg	neg	2.5
WNb 293	4.60 (± 0.22)	0.91 (± 0.04)	41.66 (± 1.80)	0.02 (± 0.00)	1.000 (± 0.00)	0.003	0.21	1.5
WNb 294	26.04 (± 1.05)	0.41 (± 0.02)	106.20 (± 4.50)	0.03 (± 0.00)	0.999 (± 0.00)	0.026	1.79	2.6
WNb 295	NB	NB	NB	NB	NB	neg	neg	2.7
WNb 296	15.26 (± 1.25)	0.51 (± 0.01)	78.58 (± 7.75)	0.03 (± 0.01)	1.000 (± 0.00)	neg	neg	2.5
WNb 297	21.40 (± 0.05)	0.93 (± 0.02)	199.00 (± 4.00)	0.08 (± 0.02)	0.999 (± 0.00)	neg	neg	1.7
WNb 298	8.94 (± 0.21)	1.11 (± 0.03)	98.70 (± 0.25)	0.02 (± 0.00)	1.000 (± 0.00)	neg	neg	0.9
WNb 299	9.90 (± 0.27)	1.04 (± 0.06)	102.53 (± 3.50)	0.04 (± 0.02)	1.000 (± 0.00)	neg	neg	2.7
WNb 300	8.43 (± 0.22)	0.91 (± 0.03)	77.08 (± 4.85)	0.07 (± 0.03)	1.000 (± 0.00)	neg	neg	2.3
WNb 301	29.40 (± 0.55)	0.47 (± 0.02)	138.00 (± 2.50)	0.04 (± 0.01)	0.999 (± 0.00)	neg	neg	1.8
WNb 302	16.12 (± 0.95)	0.63 (± 0.02)	100.85 (± 2.95)	0.03 (± 0.02)	1.000 (± 0.00)	neg	neg	2.2
WNb 303	40.51 (± 2.20)	0.63 (± 0.02)	253.45 (± 5.50)	0.11 (± 0.01)	0.999 (± 0.00)	neg	neg	2.3
WNb 304	5.44 (± 0.40)	1.09 (± 0.01)	58.98 (± 3.85)	0.02 (± 0.00)	1.000 (± 0.00)	neg	neg	1.5
WNb 305	5.76 (± 0.17)	0.93 (± 0.02)	53.40 (± 2.75)	0.02 (± 0.01)	1.000 (± 0.00)	neg	neg	0.9
WNb 306	10.40 (± 0.00)	0.82 (± 0.01)	85.00 (± 1.15)	0.03 (± 0.00)	1.000 (± 0.00)	neg	neg	0.9
WNb 307	7.29 (± 0.15)	0.89 (± 0.01)	64.74 (± 0.75)	0.04 (± 0.02)	1.000 (± 0.00)	neg	neg	1.8
WNb 308	6.57 (± 0.90)	1.08 (± 0.01)	71.17 (± 9.55)	0.02 (± 0.01)	1.000 (± 0.00)	neg	neg	2.4
WNb 309	2.68 (± 0.07)	1.19 (± 0.02)	31.90 (± 0.35)	0.02 (± 0.00)	1.000 (± 0.00)	neg	neg	1.4
WNb 310	2.50 (± 0.11)	1.06 (± 0.02)	26.43 (± 0.75)	0.02 (± 0.00)	1.000 (± 0.00)	neg	neg	2.6
WNb 311	NB	NB	NB	NB	NB	neg	neg	2.2
WNb 312	10.60 (± 4.05)	0.57 (± 0.03)	59.00 (± 19.85)	0.01 (± 0.00)	0.999 (± 0.00)	neg	neg	1.0
WNb 313	41.00 (± 5.40)	0.90 (± 0.11)	365.00 (± 6.00)	0.04 (± 0.03)	0.973 (± 0.01)	neg	neg	2.3
WNb 314	62.10 (± 11.95)	0.71 (± 0.08)	429.00 (± 34.50)	0.06 (± 0.05)	0.974 (± 0.01)	neg	neg	2.3
WNb 315	46.90 (± 0.80)	0.78 (± 0.02)	364.00 (± 17.00)	0.03 (± 0.00)	0.997 (± 0.00)	neg	neg	2.2
WNb 316	27.72 (± 0.90)	0.72 (± 0.02)	200.30 (± 1.50)	0.05 (± 0.03)	0.999 (± 0.00)	neg	neg	1.8
WNb 317	3.15 (± 0.25)	1.09 (± 0.03)	34.20 (± 1.75)	0.07 (± 0.01)	0.999 (± 0.00)	neg	neg	1.3
WNb 318	49.05 (± 11.10)	2.15 (± 0.38)	1026.20 (± 68.00)	0.10 (± 0.02)	0.990 (± 0.00)	neg	neg	1.7
WNb 319	95.20 (± 17.75)	1.64 (± 0.17)	1530.00 (± 130.00)	0.39 (± 0.09)	0.967 (± 0.01)	neg	neg	1.0
WNb 320	16.10 (± 1.70)	1.34 (± 0.10)	213.60 (± 6.50)	0.09 (± 0.03)	0.997 (± 0.00)	neg	neg	1.4
WNb 293-Fc	7.51 (± 0.95)	1.01 (± 0.02)	75.77 (± 7.90)	0.01 (± 0.00)	0.998 (± 0.00)	0.0035	0.04	15.0
WNb 294-Fc	36.88 (± 9.30)	0.45 (± 0.01)	163.75 (± 38.00)	0.02 (± 0.01)	0.999 (± 0.00)	0.045	0.57	15.0

^*a*^ Binding data including mean ± SD values for *K*_*D*_, *k*_*a*_, *k*_*d*_, *X*^2^, and *R*^2^ values as measured by bio-layer interferometry. Neutralization values shown in milligram per milliliter and nanomolar for neutralization in MNV assays. Expression yields shown in mg for 250 mL periplasmic expression in *E. coli*.

^*b*^ NB, non-binding.

Using bio-layer interferometry, we confirmed that 40 out of 45 nanobodies bound to recombinant OC43 S1_B+C_ with *K*_D_ ranging from 1.55 to 148.70 nM (Fig. 1C; Table 2; Fig. S3). The irrelevant nanobody controls WNb 15 and WNb 36 which have specificity to SARS-CoV-2 had no detectable binding to OC43 S1_B+C_ (42). WNb 293 and WNb 294 bound with *K*_D_ 4.60–26.04 nM, respectively (Table 2). To determine if WNb 293 and WNb 294 recognize distinct antigenic sites, we performed a limited nanobody competition experiment with WNb 293 and WNb 294 together with the top 7 candidates based on nanobody affinities to OC43 S1_B+C_ (Fig. 1D). We observed that the nine nanobodies bound to the OC43 S1_B+C_ in two major groups with WNb 317 bound to a distinct epitope bin, and the other cluster containing WNb 293, WNb 294, and the other six nanobodies.

### Prophylactic administration of WNbFc fusions reduces viral loads in mice

**Fig 2:**
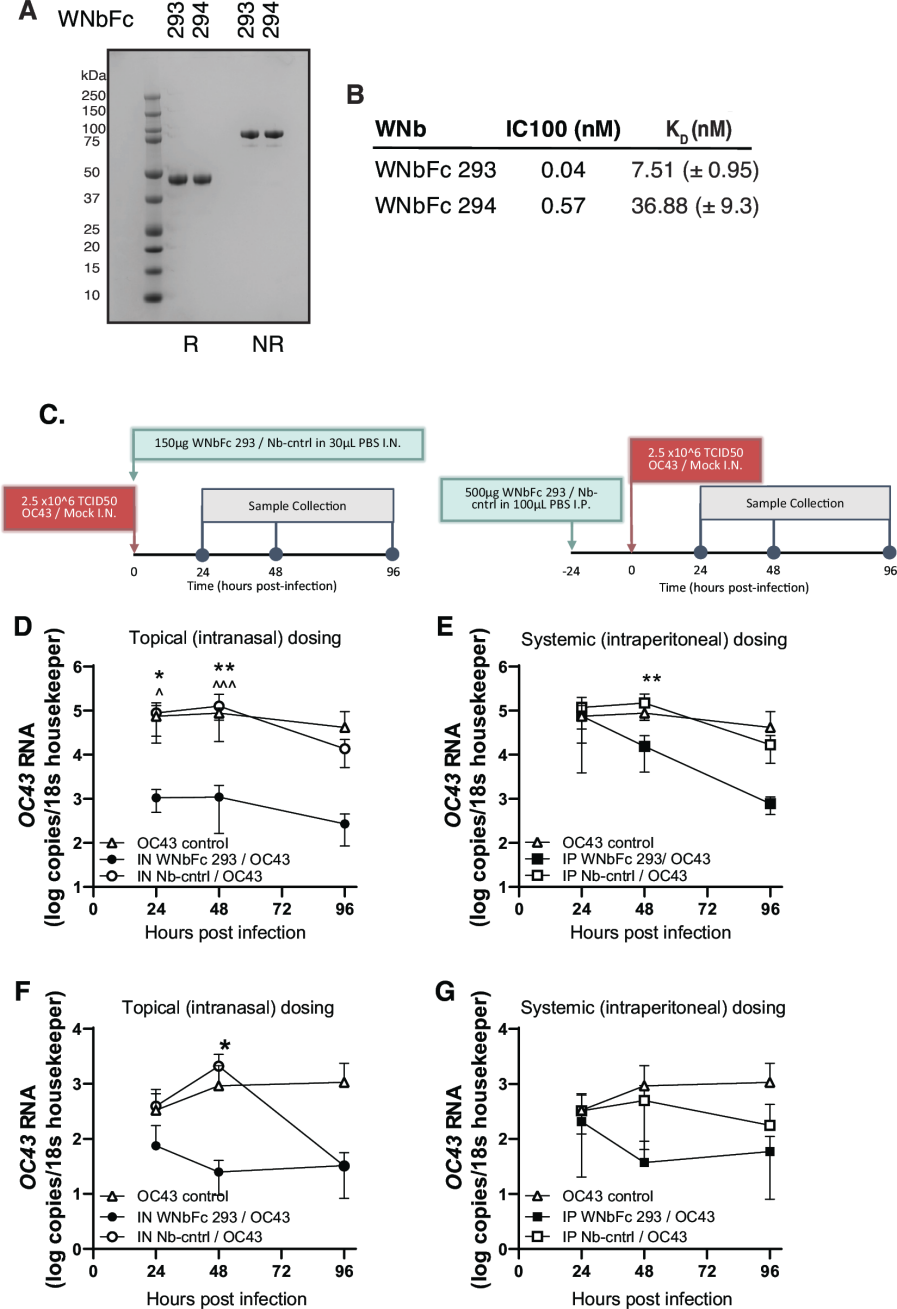
Antibody affinities and neutralization potencies of bivalent nanobody-Fc fusions. (**A**) Purified WNbFc 293 and WNbFc 294 migration on an SDS-PAGE under non-reducing (NR) and reducing (R) conditions. Molecular weight in kilodalton is shown on the left. (**B**) Neutralization of OC43 using bivalent nanobody-Fc fusions. For MNV, the values are the geometric mean of *n* = 2–4 biological replicates. Bio-layer interferometry affinity measurements with immobilized WNbFc fusion and OC43 S1_B+C_ in solution. Corresponding mean + SEM *k*_*d*_ values are indicated. (**C**) Mouse models of infection. Nasal tissue lysate viral load was determined by qPCR (normalized to 18s house-keeper) following (**D**) intranasal and (**E**) intraperitoneal dosing (*n* = 6). Viral load in nasal wash was determined by qPCR following (**F**) intranasal and (**G**) intraperitoneal dosing (*n* = 6). *P < 0.05, **P < 0.01 (WNbFc 293 compared to Nb-cntrl), ^*P* < 0.05, ^^^*P* < 0.001 (WNbFc 293 compared to untreated control) by two-way ANOVA with Holm-Sidak correction for multiple comparisons.

The two lead nanobodies WNb 293 and WNb 294 were fused to the Fc domain of human IgG1 to allow bivalent binding and to prevent rapid clearance *in vivo*. The resulting WNbFc 293 and WNbFc 294 fusions were purified as dimers (Fig. 2A). WNbFc 293 and WNbFc 294 bind to OC43 S1_B+C_ with *K*_D_ 7.51 and 36.88 nM, respectively (Fig. 2B; Table 2). WNbFc 293 and WNbFc 294 neutralize hCoV-OC43 with increased potency compared to their monomer counterpart at 0.04 and 0.57 nM, respectively (Fig. 2B). To determine neutralizing activity *in vivo* with topical administration in the upper respiratory tract, a mouse model of OC43 infection was developed (52) based on prior work (53, 54). In this model, the virus replicates 1–2 logs above input virus, peaking at day 2 post infection and is therefore suitable for studying interventions that aim to block infection. Mice were either dosed with WNbFc 293 intranasally at the same time as hCoV-OC43 infection (Fig. 2C, left) or intraperitoneally 24 hours before infection (Fig. 2C, right) to determine if systemic administration inhibits hCoV-OC43 replication in the upper respiratory tract. Infected controls were untreated (OC43 control) or dosed either intranasally or intraperitoneally with a non-specific nanobody-Fc fusion (Nb-cntrl). Nasal turbinates and nasal washes were collected at indicated times after infection to assess viral load (Fig. 2C).

Intranasal treatment with WNbFc 293 rapidly reduced viral load in nasal turbinates (84-fold) compared with both control groups by 24 hours post infection with the peak effect observed at 48 hours post infection (115-fold reduction) (Fig. 2D). Systemic intraperitoneal WNbFc 293 administration also reduced turbinate viral load (approx. 10-fold) compared to Nb-cntrl-treated mice at 48 hours post infection (Fig. 2E). To gain further insight into effect on suppressing infection viral load in nasal wash, virus shedding was also assessed. Mice treated intranasally had reduced viral shedding from 24 hours post infection which was significant when compared to Nb-cntrl-treated mice (approx. 84-fold) by 48 hours (Fig. 2F). Dosing intraperitoneally also reduced virus shedding in the upper respiratory tract, and this was significant (18-fold) by 96 hours post infection compared to OC43-infected control group (Fig. 2G).

### Structural characterization of neutralizing hCoV-OC43 nanobody

**Fig 3:**
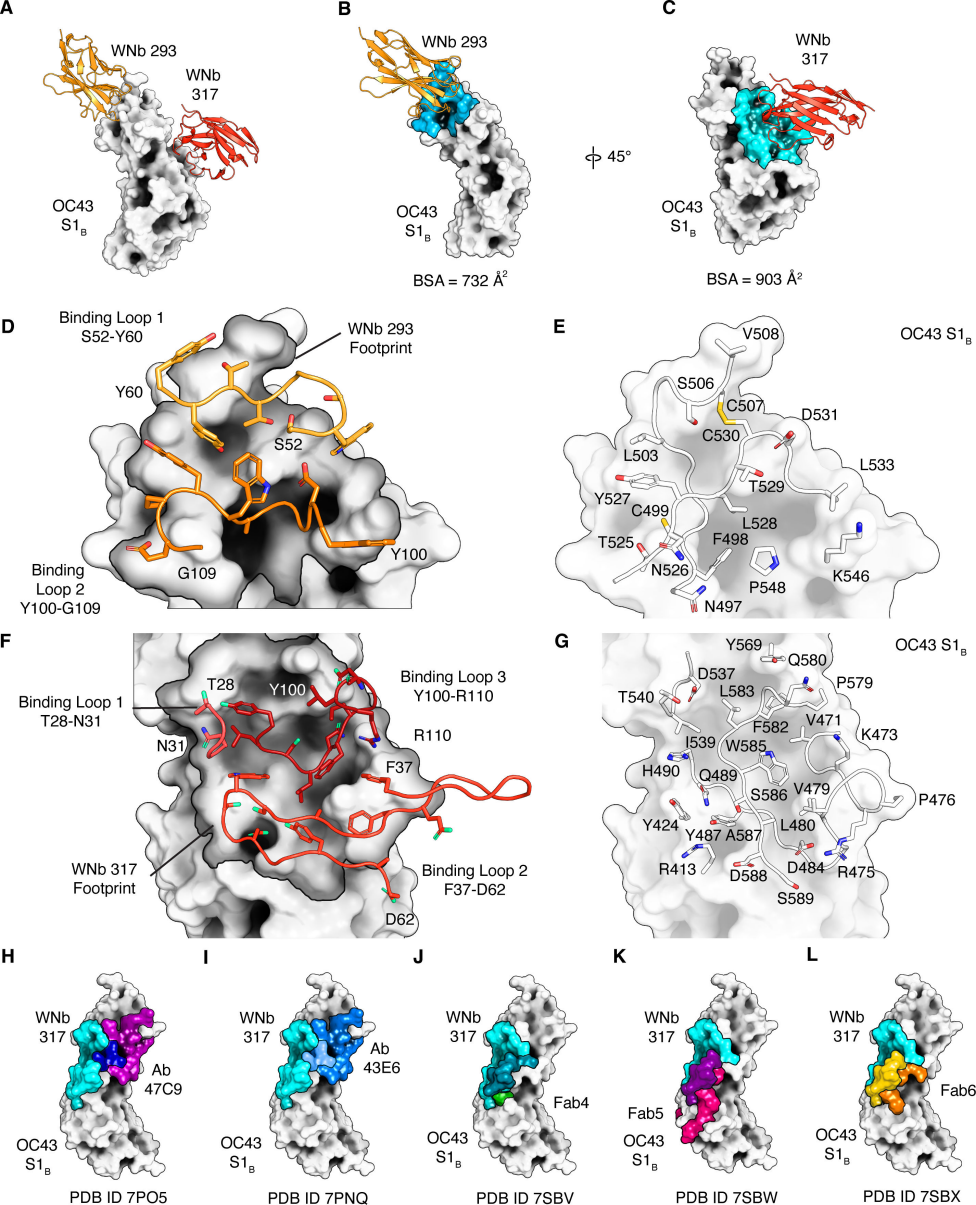
X-ray structure of the OC43 S1_B_-WNb 293-WNb 317 complex. The structure of the OC43 S1_B_ domain (gray) was solved in a dual complex with (**A**) neutralizing nanobody WNb 293 (gold) and non-neutralizing nanobody WNb 317 (orange-red). (**B**) The footprint of WNb 293 (gold)-binding residues on the OC43 S1_B_ is shown in blue and (**C**) that of WNb 317 (orange-red) in cyan (residues involved in binding are highlighted). (**D**) WNb 293 loops interacting with OC43 S1_B_ are shown in gold (binding loop 1: CDR2) and orange (binding loop 2: CDR3) over the OC43 S1_B_-binding footprint (dark gray, black outline). (**E**) OC43 S1_B_ surfaces are shown with the amino acid residues involved in binding WNb 293. (**F**) WNb 317 loops interacting with OC43 S1_B_ are shown in coral (binding loop 1: CDR1), dark orange (binding loop 2: FR2 and CDR2), and red (binding loop 3: CDR3) over the OC43 S1_B_-binding footprint (dark gray, black outline). (**G**) OC43 S1_B_ surfaces are shown with the amino acid residues involved in binding WNb 317. (**H–L**) Residue-binding footprints of mAbs overlapping the WNb 317-binding site are represented on the OC43 S1_B_ surface. The WNb 317 footprint is shown in cyan, overlapping with (**H**) mAb 47C9 (purple, blue overlap). (**I**) 43E6 (blue, light blue overlap). (**J**) Fab4 (green, teal overlap). (**K**) Fab5 (pink, purple overlap). (**L**) Fab6 (orange, yellow overlap). Interaction surfaces, hydrogen bonds and salt bridges were determined by PDBePISA.

To visualize how WNb 293 bound to OC43 S1_B_, we incubated WNb 293 and OC43 S1_B_ at a ratio of 1.2:1 for 1 hour at room temperature and purified the complex by size exclusion chromatography (SEC). Unfortunately, none of our initial crystallization hits were suitable for high resolution diffraction. To improve crystallization success, we added WNb 317 to the WNb 293-OC43 S1_B_ complex. WNb 317 is a non-neutralizing nanobody that binds to OC43 S1_B_ with *K*_*D*_ 3.15 nM and to a non-overlapping epitope compared to WNb 293 (Fig. 1B and D; Table 2). We determined the crystal structure of WNb 293-WNb 317 OC43 S1_B_ to 2.90 Å (Fig. 3A; Fig. S4). The complex was solved in the P2_1_2_1_2_1_ space group with three complexes in the asymmetric unit (Table 3). We found that WNb 293 contacts the OC43 S1_B_ at the top left of the subunit, while WNb 317 contacts toward the top center of S1_B_ when oriented in the open spike conformation with the S1_B_ connections to the S1_C_ and S1_D_ subunits as “down”(Fig. 3B and C). Overlaying the OC43 S1_B_ with that of SARS-CoV-2 reveals that both of our nanobodies bind in OC43s structurally unique RBM region.

**TABLE 3:** Data collection and refinement statistics for OC43 S1_B_ WNb 293 WNb 317 complex^*b*^

	OC43 S1_B_ WNb 293-WNb 317 (PDB 8TZU)
Data collection	
Space group	*P* 2_1_ 2_1_ 2_1_
Cell dimensions	
*a*, *b*, *c* (Å)	98.91, 150.61, 155.11
*a, b, g* (°)	90, 90, 90
Resolution (Å)	49.46–2.90 (3.06–2.90)^*a*^
*R*_merge_	0.038 (1.249)
*I/*σ(*I*)	11.9 (1.4)
CC_1/2_	0.995 (0.601)
Completeness (%)	100.0 (100.0)
Redundancy	6.9 (6.8)
Refinement	
Resolution (Å)	49.46–2.90
No. of reflections	52,029
*R*_work_/*R*_free_ (%)	23.83/26.80
No. of atoms	
Protein	10,766
Ligand/ion	183
Water	77
*B* factors	
Protein	63.84
Ligand/ion	90.43
Water	74.84
R.m.s. deviations	
Bond lengths (Å)	0.0060
Bond angles (°)	0.9890
Validation	
MolProbity score	1.75
Clashscore	10.4
Poor rotamers (%)	0.86
Ramachandran plot	
Favored (%)	96.1
Allowed (%)	3.75
Disallowed (%)	0.15

^*a*^ Values in parentheses are for highest-resolution shell.

^*b*^ X-ray diffraction data were collected on single crystals.

WNb 293 binds primarily via the CDR2 and CDR3 loops, with a buried surface area of 732 Å^2^ (Fig. 3D and E). A total of 19 WNb 293 residues form the binding interface, including 19 hydrogen bonds (Table S1) (Fig. S4A and B). S1_B_ contacts WNb 293 with binding loop 1 (CDR2 and surrounding residues from residues 52 to 60) and the binding loop 2, which consists of residues 100–109 within the CDR3 (Fig. 3D; Table S1). These WNb 293 contact residues bind a continuous footprint on the S1_B_ surface covering residues 497–499, 503–508, 524–533, and 546–548 (Fig. 3E; Table S1).

WNb 317 contacts the S1_B_ via binding loop 1 (residues 28–31 with CDR1), binding loop 2 (residues 37–62 in FR1, FR2, and CDR2) and binding loop 3 (residues 100–110 within CDR3) with a total buried surface area of 903 Å^2^ (Fig. 3F and G). The WNb 317-binding epitope consists of 28 WNb 317 residues including 21 hydrogen bonds and 1 salt bridge (Fig. S4C and D; Table S1). Briefly residues 28–32, 37, 45–61, 65, and 100–110 of WNb 317 contact a continuous conformational footprint on the S1_B_ consisting of residues 413, 424, 470–480, 484, 487, 489–490, 536–540, 566, 569, and 579–589 (Fig. 3G).

Using SEC, we were able to obtain a WNb 317 OC43 spike complex but unable to complex WNb 293 with OC43 spike (Fig. S5A and B). However, there is no obvious steric hinderance for WNb 293-OC43 spike binding when overlayed on a cryo-EM map of the closed conformation (12–15, 17, 55) (Fig. S5C). There are three substitutions in WNb 293 footprint on OC43 between the isolates used here for OC43 S1_B_ and OC43 spike (GenBank AAT84362.1 and GenBank AIL49484.1, respectively). Of these, the D531 sidechain in the OC43 S1_B_ is involved in multiple hydrogen bonds to the WNb 293 CDR2 in the crystal structure, which may be abrogated by its substitution to histidine.

Five antibodies, 47C9 (PDB 7P05), 43E6 (PDB 7PNQ), Fab4 (PDB 7SBV), Fab5 (PDB 7SBW), and Fab6 (PDB 7 SBX), bind to epitopes that partly overlap that of WNb 317 on the OC43 S1_B_ domain (PDBePISA calculation) (33, 40) (Fig. 3H through L). Two of these antibodies, 43E6 and 47C9, were tested for *in vitro* neutralization and are potently neutralizing, while the remaining three antibodies were not tested for *in vitro* neutralization (33). The overlapping interface of 43E6 and 47C9 with WNb 317 covers 4 of 18 OC43 S1_B_ contact residues and 4 of 19 OC43 S1b contact residues, respectively (33). The binding epitope of a further neutralizing antibody 37F1 (EMD 13564) was partly defined by low-resolution electron microscopy (4.4 Å) and overlaps with residues 497–508 bound by the CDR2 and CDR3 loops of WNb 293 (Fig. S3F) (33).

## DISCUSSION

Here, immunization of an alpaca with recombinant OC43 protein enabled the identification of two potent neutralizing nanobodies against hCoV-OC43, WNb 293, and WNb 294. Furthermore, when fused to the Fc domain of human IgG1, the WNb 293-Fc fusion retained high-affinity binding *K*_*D*_ 7.51 nM with potent neutralizing activity against hCoV-OC43 as low as 0.04 nM. To the best of our knowledge, the WNb 293-Fc fusion is the first nanobody Fc fusion shown to prevent hCoV-OC43 virus infection in mice. Our crystal structure of WNb 293 bound to OC43 S1_B_ reveals a neutralizing epitope with unknown function that binds a separate domain to that interacting with sialoglycans in host cell entry (Fig. 3A; Fig. S1A).

We determined the effectiveness of WNbFc 293 as an anti-viral drug—delivered either topically intranasally to model a nasal spray-based treatment or systemically intraperitoneally to determine if indirect treatment could provide protection in the upper respiratory tract. Intranasal treatment at the time of infection was highly effective reducing viral load in nasal tissue and virus shedding in nasal wash and clearly supports the development of a nanobody-based nasal spray. Further work is now needed to determine the window of anti-viral treatment effect pre- and post-infection. Systemic delivery was also effective at suppressing viral replication in the upper respiratory tract. The magnitude of reduction (10-fold compared to controls) was less than that observed with intranasal treatment (>100-fold). Further studies will build on this proof-of-concept data to determine the duration of anti-viral activity of CoV-targeting nanobody-Fc fusions in the upper respiratory tract following intranasal topical delivery versus systemic treatment intraperitoneally.

Two neutralizing nanobodies were identified from our immunized library, WNb 293 and WNb 294 (Fig. 1A; Table 1). These nanobodies share a high degree of homology with 92% sequence identity. Both the CDR1 and CDR2 sequences for these nanobodies are identical, with six changes in the framework regions and four substitutions in the CDR3 loop (Table 1). Despite this sequence conservation, the neutralizing potency of WNb 293 is more than eightfold higher than for WNb 294 (Fig. 1B). Our structural analyses of WNb 293 with OC43 S1_B_ do not provide definitive explanations for the difference in potency. The OC43 S1_B_-WNb 293-WNb 317 structure reveals that none of the framework substitutions are close to the S1_B_-binding interface, suggesting that these framework changes are not responsible for the difference in potency. Only two of the four CDR3 substitutions are involved in direct OC43 S1_B_ interactions, G100W and F106Y (WNb 294 residue, position, WNb 293 residue). The hydroxyl introduced by the F106Y substitution does not bind to OC43 S1_B_ and is unlikely to have any impact on the interaction. The WNb 293 W100 contacts K546 OC43 S1_B_ via the sidechain which would not be possible with G100 in WNb 294 and may impact WNb 293 binding to OC43 S1_B_ (Fig. 3D; Fig. S3A). The remaining two residues substituted in the CDR3 loop, E99L, and A111S do not directly contact OC43 S1_B_. However, these CDR3 loop substitutions result in additional sidechain-mediated interactions within the CDR3 loop and between the surrounding nanobody framework regions. These may impact the conformation and stabilization of the CDR3 loop as a whole and alter the interaction between OC43 S1_B_ and the nanobody, though this requires further mutational analyses.

Though sequence information is extremely limited for hCoV OC43 compared with SARS-CoV-2, analysis of 213 unique OC43 S1_B_ sequences obtained from the Bacterial and Viral Bioinformatics Resource Center (https://www.bv-brc.org) revealed considerable variation within the domain (Fig. S1B and C). Within the WNb 293 footprint on the S1_B_, several residues display considerable variation (Fig. S5E). In particular, the loop region from 503 to 508 contains several highly variable residues with the direct WNb 293 contact position 505 being conserved in only 57% of our 213 identified S1_B_ sequences (Fig. S1B and C). Along with the previously identified contact residue mutation D531H in strain 1783A_10 (GenBank AIL49484.1), this suggests that, similar to SARS-CoV-2, neutralization may be strain specific for some antibodies and nanobodies.

We identified WNb 317 as a non-neutralizing nanobody that binds to OC43 S1_B_ with *K*_*D*_ 3.15 nM. Both WNb 293 and WNb 317 were found to bind to a structurally unique region of the OC43 S1_B_ that corresponds to the RBM in other hCoV S1_B_ domains (Fig. 3A through C). A large collection of RBM-targeting antibodies have been identified against SARS-CoV-2 and were grouped into class 1 and class 2 depending on the epitope targeted; high-affinity antibodies in these classes were found to be potently neutralizing (56–58). The majority of antibodies in these classes neutralized only a narrow range of SARS-CoV-2 VoC due to the rise of escape mutations in the RBM (58); however, there are some reports of broadly neutralizing antibodies targeting these epitopes (59, 60).

Using cryo-EM, Wang et al. and Bangaru et al. structurally characterized five human antibodies that partly overlap with the WNb 317-binding site on OC43 (Fig. 3H through L) (33, 40) as well as determining a low-resolution structure of an antibody which may overlap the WNb 293 site (Fig. S5D). Two of the five WNb 317 antibodies, 43E6 and 47C9, display *in vitro* neutralizing activity (IC_50_ = 0.105 and 0.228 µg/mL, respectively) using virus neutralization assays, while the remaining three have not been tested (33). Unfortunately, direct structural comparison is somewhat hindered by difference in resolution of the neutralizing antibody structures, 47C9 and 43E6 at 3.9 and 3.7 Å, respectively, compared with 2.9 Å for WNb 317. However, their interfaces have a broadly similar surface area at 903, 766.5, and 714 Å^2^ for WNb 317, 47C9, and 43E6 respectively (PDBePISA). The 47C9 and 43E6 heavy chains partly overlap with the binding epitope for WNb 317 (Fig. 3H and I; Table S1). However, 26 of 31 OC43 S1_B_ contact residues with WNb 317 are unique to the WNb 317 interaction with no neutralizing antibodies identified having a more similar epitope to WNb 317.

We speculate that the mechanism of neutralization for 47C9 and 43E6 might be due to the unique portions of their epitopes compared to WNb 317 which could incorporate part of a putative receptor-binding site. Differences in size and orientation between 47C9/43E6 antibodies at 150 kDa and the WNb 317 nanobody at just 14 kDa may also allow the larger antibodies to sterically hinder an OC43 S1_B_ interaction with a putative receptor at an adjacent site.

Alternatively, sequence variation between OC43 isolates may be responsible for the lack of neutralization in our MNV assay. Similarly to the WNb293 footprint, the WNb 317-binding interface includes regions containing considerable variation. The region 470–480 has several variable positions, particularly 480 which is only conserved in 59% of our 213 isolates (Fig. S1B and C), and forms part of a hydrophobic patch, which coordinates WNb 317 CDR2 and CDR3 binding (Fig. S4G; Fig. S5E). Screening for neutralization against a broader range of clinical isolates may provide additional information on the neutralizing capacity of this epitope.

Wang et al. also identified neutralizing antibodies 56E10, 45B9, and 65A11 that bind to a cryptic epitope which is unavailable in the closed spike conformation (33). Though they were unable to structurally characterize this epitope, HDMX studies showed that these antibodies bound to residues 399–406, 417–421, and 537–547 (33). Interestingly, WNb 293 contacts 546 in this proposed cryptic region, and we were unable to complex WNb 293 with using a prefusion-stabilized OC43 spike which is in the closed conformation (Fig. S3C and D), though this is likely due to sequence variation between viral isolates used for our recombinant spike and S1_B_ domains (GenBank AAT84362.1 and GenBank AIL49484.1, respectively). Further structural insight or alternative approaches are required to fully determine if WNb 293 occupies part of a cryptic epitope on OC43 spike.

Nanobody and antibody cocktails targeting non-overlapping epitopes on the coronavirus receptor-binding domains have shown promise in preventing occurrence of resistance mutation. Combining nanobodies and conventional antibodies against hCoV-OC43 may be advantageous for controlling more highly infectious variants and reducing the potential for virus escape mutations to develop. Proteinaceous receptors for all other hCoVs have been identified (12–17, 61) The identification of a proteinaceous entry receptor for OC43 could help to provide insight into the neutralizing mechanisms of these nanobodies that do not bind at the OC43 sialoglycan-binding site but do bind at the S1_B_ domain, which is the protein receptor-binding site in all other hCoV.

## MATERIALS AND METHODS

### OC43 recombinant protein expression and purification

For immunization of alpacas and *in vitro* assays, two recombinant OC43 constructs were designed consisting of the S1_B_ and S1_C_ domains [OC43 S1_B+C_, amino acid (aa) 321–675, Uniprot P36334] and the S1_B_ domain alone (OC43 S1_B_, aa 331–608), respectively. Recombinant OC43 S1_B+C_ contained a C-terminal tobacco etch virus (TEV) protease site to allow cleavage of the hexahistidine tag, while OC43 S1_B_ has a C-terminal hexahistidine tag only.

Both constructs were expressed in Expi293F suspension cells (ThermoFisher), which were maintained in suspension at 37°C, ≥80% relative humidity and 8% CO_2._ Transfection was performed using the ExpiFectamine 293 Transfection Kit following the manufacturer’s protocol (Thermo-Fisher/Gibco #A14525). After 6 days, the supernatant was collected by centrifugation and filtered through a 0.22-µm filter. The proteins were individually purified by loading the supernatant on a 5-mL HisTrap Excel HP column (Cytiva Cat# 17371206) and eluted over Ni-NTA IMAC chromatography gradient from 0% to 100% B (buffer A: 5 mM imidazole pH 7.5, 100 mM NaCl DPBS; buffer B: 400 mM imidazole pH 7.5, 100 mM NaCl DPBS). Elution fractions containing the respective proteins were pooled and concentrated using a 10K MWCO Amicon Ultra-15 centrifugal filter unit (Merck Millipore #UFC901096). A second step purification was performed on Superdex 200 10/300 Increase GL size exclusion chromatography column (Cytiva Cat# 28990944) pre-equilibrated with DPBS pH 7.5 buffer for functional studies or 20 mM HEPES 150 mM pH 7.5 for crystallographic studies.

To remove the hexahistidine tag from OC43 S1_B_, TEV protease was added to purified protein and incubated overnight at 4°C. The solution was applied to an Ni-NTA IMAC column to capture any residual His-tagged OC43 S1_B_ and His-tagged TEV protease. The flow through containing untagged hCoV-OC43 S1_B_ was applied to Superdex 75 10/300 Increase size exclusion chromatography column (Cytiva #29148721) pre-equilibrated in 1× DPBS pH 7.5 for a final purification.

### Alpaca immunization

One alpaca was immunized six times with approximately 200 µg of recombinant OC43 S1_B+C_ protein on days 0, 14, 21, 28, 35, and 42. The adjuvant used was GERBU FAMA. Immunization and handling of the alpacas for scientific purposes were approved by Agriculture Victoria, Wildlife and Small Institutions Animal Ethics Committee, project approval No. 26–17. Blood was collected 3 days after the last immunization for the preparation of lymphocytes. Nanobody library construction was carried out according to established methods as described (62). Briefly, alpaca lymphocyte mRNA was extracted and amplified by RT-PCR with specific primers to generate a cDNA nanobody library. The library was cloned into a pMES4 phagemid vector containing 10^4^ unique nanobodies, amplified in *E. coli* TG1 strain, and subsequently infected with M13K07 helper phage for recombinant phage expression.

### Isolation of OC43 S1_B+C_ nanobodies

Biopanning for recombinant OC43 S1_B+C_ nanobodies using phage display was performed as previously described with following modifications (42). Phages displaying OC43 S1_B+C_ -specific nanobodies were enriched after two rounds of biopanning on streptavidin-coated dynabeads (Invitrogen) coated with 200 pMol of biotinylated recombinant protein. After the second round of panning, we screened 376 clones from both round 1 and round 2 by ELISA and observed a 90% positive hit rate, and 676 clones were selected for further analysis. Positive clones were sequenced and annotated using the International ImMunoGeneTics database (IMGT) and aligned in Geneious Prime 2020.2.4. A total of 45 distinct nanobody clonal groups were identified based on at least one amino acid difference in the complementary determining region 3.

### Nanobody expression and purification

Nanobodies were expressed in *Escherichia coli* WK6 cells (63). Bacteria were grown in 250 mL Terrific Broth supplemented with 0.1% (wt/vol) glucose and 100 ug/mL carbenicillin at 37°C to an OD_600_ of 0.7, induced with 1 mM IPTG, and grown overnight at 28°C for 16 hours. Cell pellets were harvested and resuspended in 20% (wt/vol) sucrose, 10 mM imidazole pH 7.5, 150 mM NaCl, DPBS and incubated for 15 min on ice. Furthermore, 5 mM EDTA pH 8.0 was added and incubated on ice for 20 min. After this incubation, 10 mM MgCl_2_ was added to prevent nickel ion-EDTA chelation, periplasmic extracts were harvested by centrifugation, and the supernatant was loaded onto a 1-mL HisTrap Excel HP column (Cytiva Cat# 17371205). The nanobody was eluted with 400 mM imidazole pH 7.5, 100 mM NaCl, DPBS. The appropriate fractions were concentrated, and buffer exchanged into sterile DPBS using 3K MWCO Amicon Ultra-15 centrifugal filter unit concentrators (Merck Millipore).

### WNbFc fusion cloning, expression, and purification

Synthetic human IgG1-Fc sequence (IDT) was cloned into the pHLSec vector via restriction sites AgeI and XhoI to generate pHLSec-Fc. Nanobody-encoding sequences were amplified by PCR using Nano15-fwd (5′- TAGACCGGTCAGGTGCAGCTGCAG-3′) and Nano18-rev (5′- CTAGCTAGCGAGGGGACGGTCACCTGG-3′) and inserted in pHLSec-Fc via AgeI and NheI to generate the respective WNbFc expression constructs. Recombinant WNbFc fusions were expressed in Expi293F HEK cells (ThermoFisher), which were maintained in suspension at 37°C, 8% CO_2_, and ≥80% relative humidity. Cells were transfected at a density of 2.5–3 × 10^6^ with 1 µg/mL of plasmid DNA/mL of expression culture and ExpiFectamine 293 reagent diluted in Opti-MEM I (Reduced Serum media) (ThermoFisher/Gibco #31985088) following the manufacturer’s protocol. At 22 hours post transfection, ExpiFectamine 293 transfection Enhancers 1 and 2 (ThermoFisher/Gibco) were added to transfected cells along with 20% lupin peptone. Five days post transfection, the supernatant was harvested by centrifugation and filtered through a 0.22-µm filter. WNbFcs supplemented MgCl_2_ (to a final concentration of 10 mM) were purified by loading the supernatant onto a 1-mL HiTrap MabSelect Prism A HP column (Cytiva Cat#17549852). WNbFcs were eluted using 100 mM citric acid pH 3.0 and neutralized with 1 M Tris-HCl pH 9.0. Prism A eluate fractions were buffer exchanged into 1× DPBS using Amicon Ultra-15 30K MWCO concentrators (Merck Millipore) prior to further purification on a Hiload 16/600 Superdex 200 pg gel filtration chromatography column (GE Healthcare) pre-equilibrated with 1× DPBS pH 7.5. Antibody concentration was determined by absorbance measurement at 280 nm using a Nanodrop, and purity was determined using SDS-PAGE.

### OC43 propagation and quantification

Original OC43 (ATCC Number VR-1558) passage history is unknown and propagated on HCT-8 cells. Initial stocks from ATCC were received at concentrations of 2.8 × 10^5^ TCID50/mL and passaged two times in MRC-5 cells to generate a working stock. MRC-5 cell lines were used for TCID50 assays using the Karber method to quantify viral load.

### OC43 microneutralization assay

The ability of nanobodies to neutralize the infectivity of 100 median tissue culture infectious doses (TCID50) of virus was assessed in a microneutralization assay as previously described (64) . Serial twofold dilutions of nanobodies starting at 1:20 were incubated with OC43 in MEM/0.5% BSA at room temperature for 1 hour. Residual virus infectivity was assessed in quadruplicate wells of MRC-5 cells, and viral cytopathic effect was read on day 7. The neutralizing antibody titer was calculated using the Reed-Muench method as previously described (64).

### Bio-layer interferometry

Nanobody affinities to recombinant OC43 S1_B+C_ were measured using an Octet RED96e instrument (ForteBio). Assays were performed at 25°C in solid black 96-well F-bottom plates (Greiner-Bio One #655209) agitated at 1,000 rpm. Kinetic buffer was composed of Gibco 1× DPBS (Life Technologies Cat# 14190144) supplemented with 0.1% (wt/vol) BSA, 0.05% (vol/vol) TWEEN-20. A 60-s biosensor baseline step was applied before nanobodies were loaded onto Ni-NTA (Octet NTA) (Sartorius) sensors by submerging sensor tips in 5 µg/mL of each nanobody until a response of 0.5 nm was obtained, then washed in kinetic buffer for 60 s. Association measurements were performed for 180 s using a twofold concentration gradient of untagged OC43 S1_B+C_ from 3 to 100 nM for higher-affinity nanobodies or 16 to 250 nM for low-affinity nanobodies. Dissociation was measured in kinetic buffer for 180 s. Sensor tips were regenerated using a cycle of 5 s in 500 mM imidazole pH 7.5 and 5 s in kinetic buffer repeated five times. Baseline drift was corrected by subtracting the shift of a nanobody-loaded sensor not incubated with cleaved OC43 S1_B+C_. Curve fitting analysis was performed with Octet Data Analysis 11.1 software (ForteBio) using a global fit 1:1 model to determine *K*_D_ values and kinetic parameters. Curves that could not be fitted were excluded from the analysis.

WNbFc antibody affinities to OC43 S1_B+C_ were measured using the above method with the following modifications. Anti-human IgG Fc capture sensor tips (Octet AHC) were used for affinity measurements. For measuring affinities against OC43 S1_B+C_, WNbFc antibodies were loaded onto sensor tips by submerging in 5 µg/mL of WNbFc antibody for 200 s or until a signal shift of 0.5 nm.

### Epitope binning

For WNb epitope binning experiment, 30 nM OC43 S1_B+C_ was pre-incubated with each nanobody at a 10-fold molar excess (300 nM) for 1 hour at RT. A 30-s baseline step was established between each step of the assay. NTA sensors were first loaded with 10 µg/mL of nanobody for 5 min. The sensor surface was then quenched by dipping into 10 µg/mL of an irrelevant nanobody (Pf12p-B9 nanobody) for 5 min (65). Nanobody-loaded sensors were then dipped into premixed solutions of OC43 S1_B+C_ and nanobody for 5 min. Nanobody-loaded sensors were also dipped into OC43 S1_B+C_ alone to determine the level of OC43 S1_B+C_ binding to immobilized nanobody in the absence of other nanobodies. Percentage competition was calculated by dividing the maximum response of the premixed OC43 S1_B+C_ and nanobody solution binding by the maximum response of OC43 S1_B+C_ binding alone, multiplied by 100.

### Inoculations

Six- to 8-week-old female BALB/c mice were obtained from Australian Bioresources (Moss Vale, Sydney, NSW). Intranasal administration of nanobodies and viral challenge were performed under light isoflurane anesthesia (66). Nanobodies were delivered at 5 mg/mL concentrations equating to 0.5 mg in a 100-µL intraperitoneal injection and 150 µg in a 30-µL intranasal administration of 1.42 × 10^6^ TCID50 units of OC43.

### RNA extraction and reverse transcription

Apical lung lobes were harvested in RNA later (Ambion) and stored at −80°C, lysed in buffer RLT (Qiagen) containing 1% betamercaptoethanol with tissue dissociation using a TissueLyser II (Qiagen). Debris was pelleted by centrifugation (10 min at 10,000 RCF), and supernatants stored at −80°C. Nasal turbinates were excised and vortexed for 30 s in RLT containing 1% beta-mercaptoethanol. Nasal turbinate debris was removed, and the lysate stored at −80°C. Apical lung lobe and nasal turbinate RNA were extracted using the miRNAeasy Kit (Qiagen) following the supplier’s protocol. Viral RNA was manually extracted from the nasal wash fluid using the QIAamp Viral RNA Mini Kit (Qiagen) following the supplier’s protocol. RNA was measured by spectrophotometry (Nanodrop), and 1,000 ng (lung), 500 ng (nasal turbinate), or 200 ng (nasal wash) of RNA was used in reverse transcription reactions with High-Capacity cDNA Reverse Transcription Kit (ABI) per manufacturers’ recommendations.

### Quantitative PCR

Quantitative PCR (qPCR) was performed on the QuantStudio 6 using TaqMan Gene Expression Master Mix (Thermo Fisher Scientific) and primer-probe combinations (Thermo Fisher Scientific) as outlined in Table 4 (67). Standards of known concentration were used for absolute quantification of genes of interest. 18S was used as the reference gene to normalize the copy numbers of the genes of interest.

**TABLE 4:** qPCR primer/probe sequences^*a*^

	Sequence (5′−3′)		
	Forward	Reverse	Probe
18S	CGCCGCTAGAGGTGAAATTCT	CATTCTTGGCAAATGCTTTCG	FAM-ACCGGCGCAAGACGGACCAGA-TAMRA
OC43	CGATGAGGCTATTCCGACTAGGT	CCTTCCTGAGCCTTAATATAGTAACC	FAM-TCCGCCTGGCACGGTACTCCCT-TAMRA

^*a*^ Primers and probes were all used at a final concentration of 5 µM. TaqMan probes all contained 5′ FAM reporter and 3′ TAMRA quencher.

### Statistical analyses

Statistical analyses were performed using GraphPad Prism software (version 9.1.2). Data spanning multiple timepoints were analyzed by two-way ANOVA or mixed-effect analysis using Holm-Sidak’s correction or Tukey’s correction for multiple comparisons unless stated otherwise in the figure legends. *P* < 0.05 was considered statistically significant.

### X-ray crystallography of the OC43 S1_B_-WNb 293-WNb 317 complex

Crystallization trials were undertaken at the Monash Macromolecular Crystallisation Facility at 20°C using 96-well sitting drop vapor diffusion plates (Greiner). Crystals of the OC43 S1_B_-WNb 293-WNb 317 complex were obtained from a solution containing 25% PEG 1500, 0.1 M DL-malic acid, MES monohydrate, Tris (MMT) pH 4.0 (Molecular Dimensions) after 3 days. Crystals were reproduced in 24-well hanging drop trays, and crystals forming in 27% PEG 1500, 0.1 M MMT pH 4.0 were used to seed into fine screens. Seeded crystals were grown in 23% PEG 1500, 0.1 M MMT pH 4.0 and flash frozen in liquid nitrogen at 100 K following equilibration in a reservoir solution containing 15% glycerol as a cryoprotectant. The MX2 beamline at the Australian Synchrotron (Melbourne, Victoria) was used to collect a data set for the OC43 S1_B_ -WNb 293-WNb 317 complex at 2.9 Å. Data were recorded using an Eiger 16M detector (Dectris) and processed using the XDS package (68). Molecular replacement was undertaken using Phaser (69, 70). A search model was generated for the OC43 S1_B_ using a portion of a previously determined cryo-electron microscopy OC43 spike structure (PDB ID 6OHW) corresponding to this domain (AA: 331–608). Nanobody search models were generated using Nanonet (71) to modify the previously determined nanobody structure (PDB ID 5LHR chain B) to better match the WNb 293 and WNb 317 sequences, respectively.

OC43 S1_B_-WNb 293-WNb 317 complex consisted of three copies of the complex in the asymmetric unit, though one copy of WNB 293 had insufficient density to support structural modeling. Refinement through iterative rounds of model building in COOT (72) and refinement in Phenix v1.19.2 (73) and Buster v2.10.4 (74) generated a final model with an *R*_obs_/*R*_free_ = 23.83/26.80. Figures were prepared using the PyMOL Molecular Graphics System, Version 2.5.2 (Schrödinger, LLC). Interfaces and interactions were analyzed using PDBePISA v1.52 (75).

## Data Availability

All data are available in the paper or in the supplemental material. The atomic coordinates and structure factors for OC43 S1_B_-WNb 293-WNb 317 have been deposited in Protein Data Bank with accession number 8TZU.

## SUPPLEMENTAL MATERIAL

Figure S1 jvi.00531-24-s0001.tif Schematic of the OC43 Spike and conservation of the S1_B_ domain among viral isolates.

Figure S2 jvi.00531-24-s0002.eps Purification of OC43 nanobodies.

Figure S3 jvi.00531-24-s0003.tif BLI-binding experiment with immobilized WNb nanobodies and in solution OC43 S1_B+C_.

Figure S4 jvi.00531-24-s0004.eps Structural details of the OC43 S1_B_-WNb 293-WNb 317 interfaces.

Figure S5 jvi.00531-24-s0005.tif Structural analysis of WNb 293 and WNb 317 binding to OC43 Spike.

Table S1 jvi.00531-24-s0006.docx Summary of interactions between OC43 RBD and nanobodies WNb 293 and WNb 317.

ASM does not own the copyrights to Supplemental Material that may be linked to, or accessed through, an article. The authors have granted ASM a non-exclusive, world-wide license to publish the Supplemental Material files. Please contact the corresponding author directly for reuse.
