# Supplementary figures and images for: Human coronavirus OC43 nanobody neutralizes virus and protects mice from infection
Source: J Virol. 2024 May 6;98(6):e00531-24. doi: 10.1128/jvi.00531-24 (PMC11237593; doi:10.1128/jvi.00531-24)

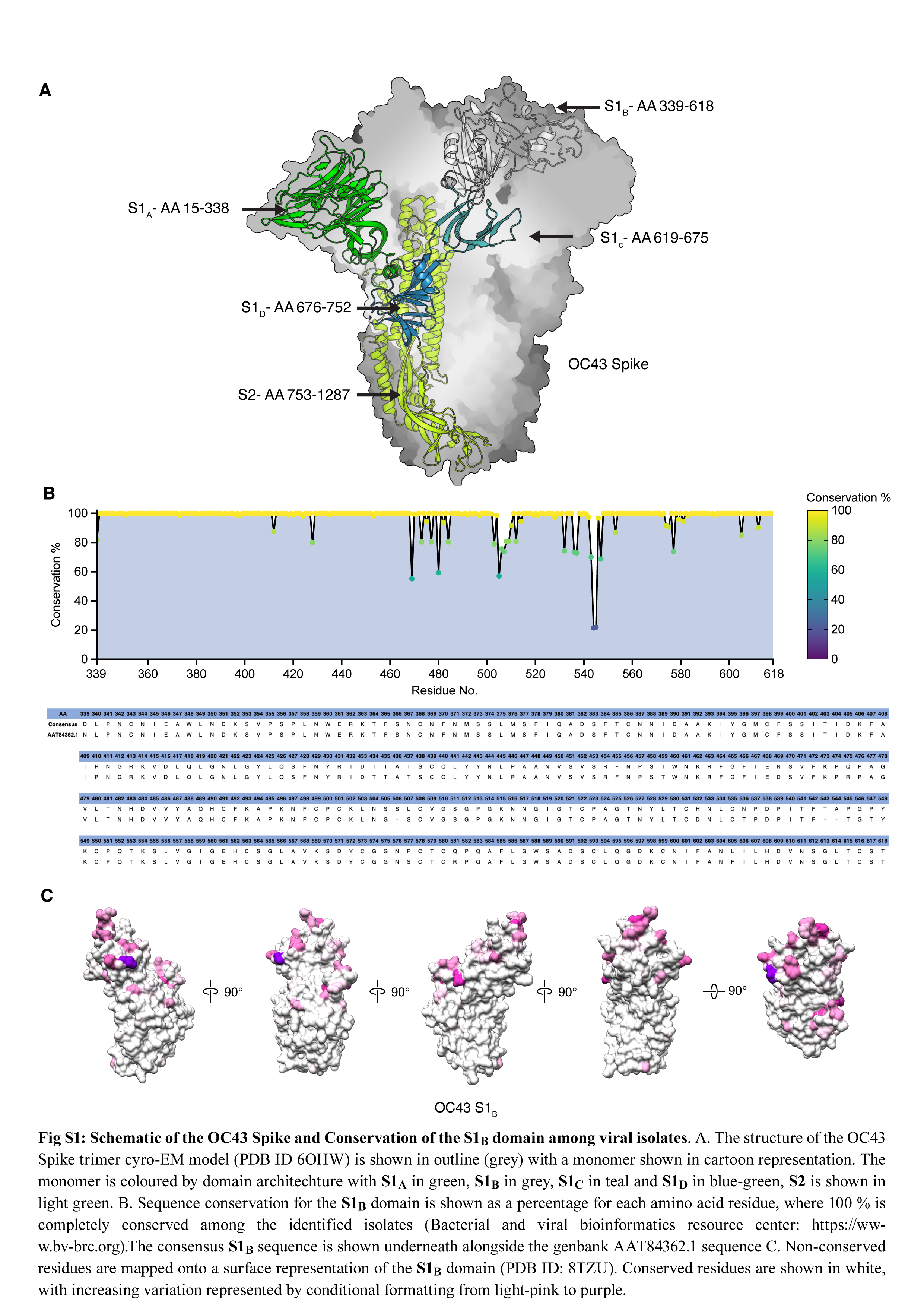

Supplement: Figure S1 — Schematic of the OC43 Spike and conservation of the S1B domain among viral isolates. [file jvi.00531-24-s0001.tif]

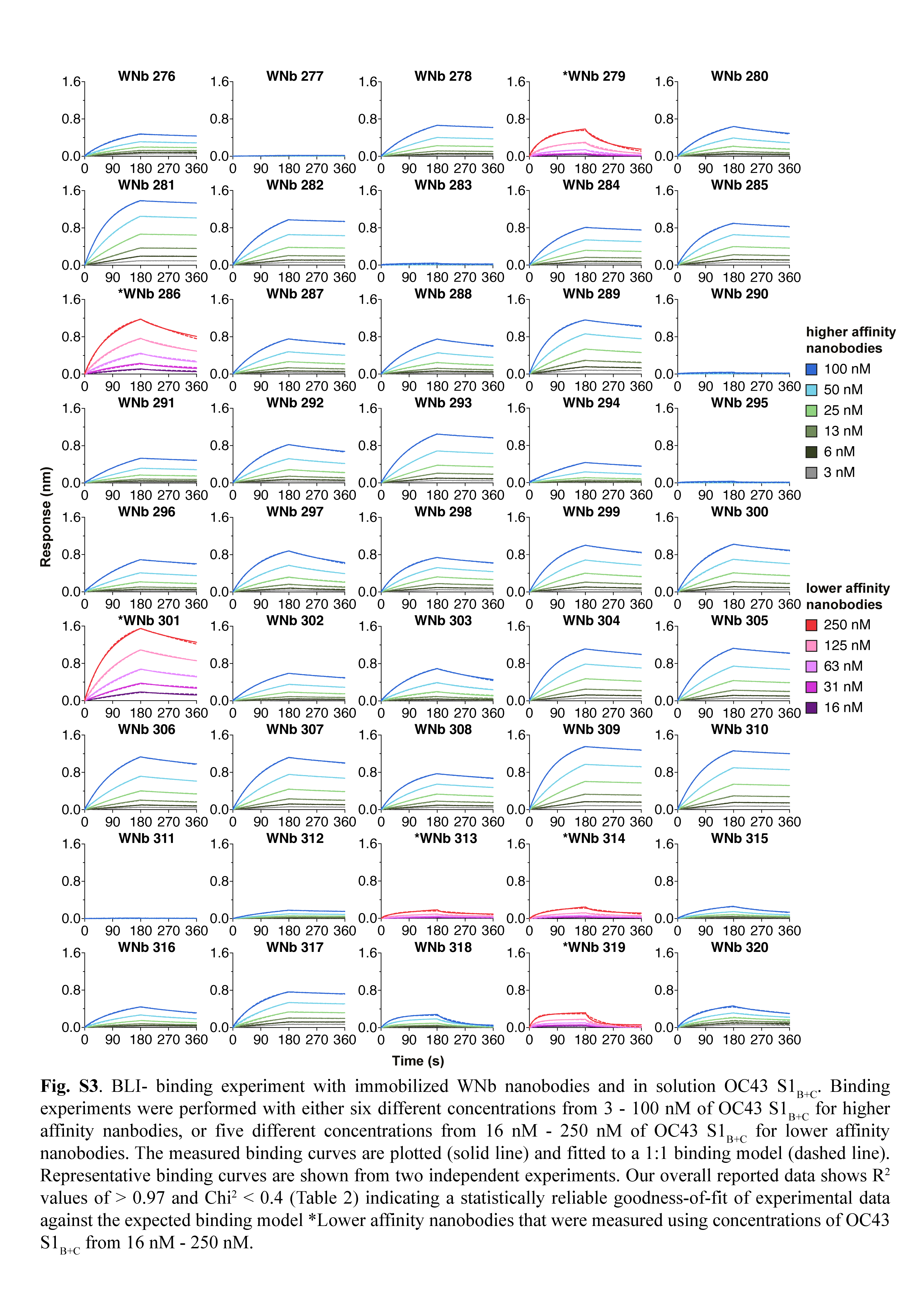

Supplement: Figure S3 — BLI-binding experiment with immobilized WNb nanobodies and in solution OC43 S1B+C. [file jvi.00531-24-s0003.tif]

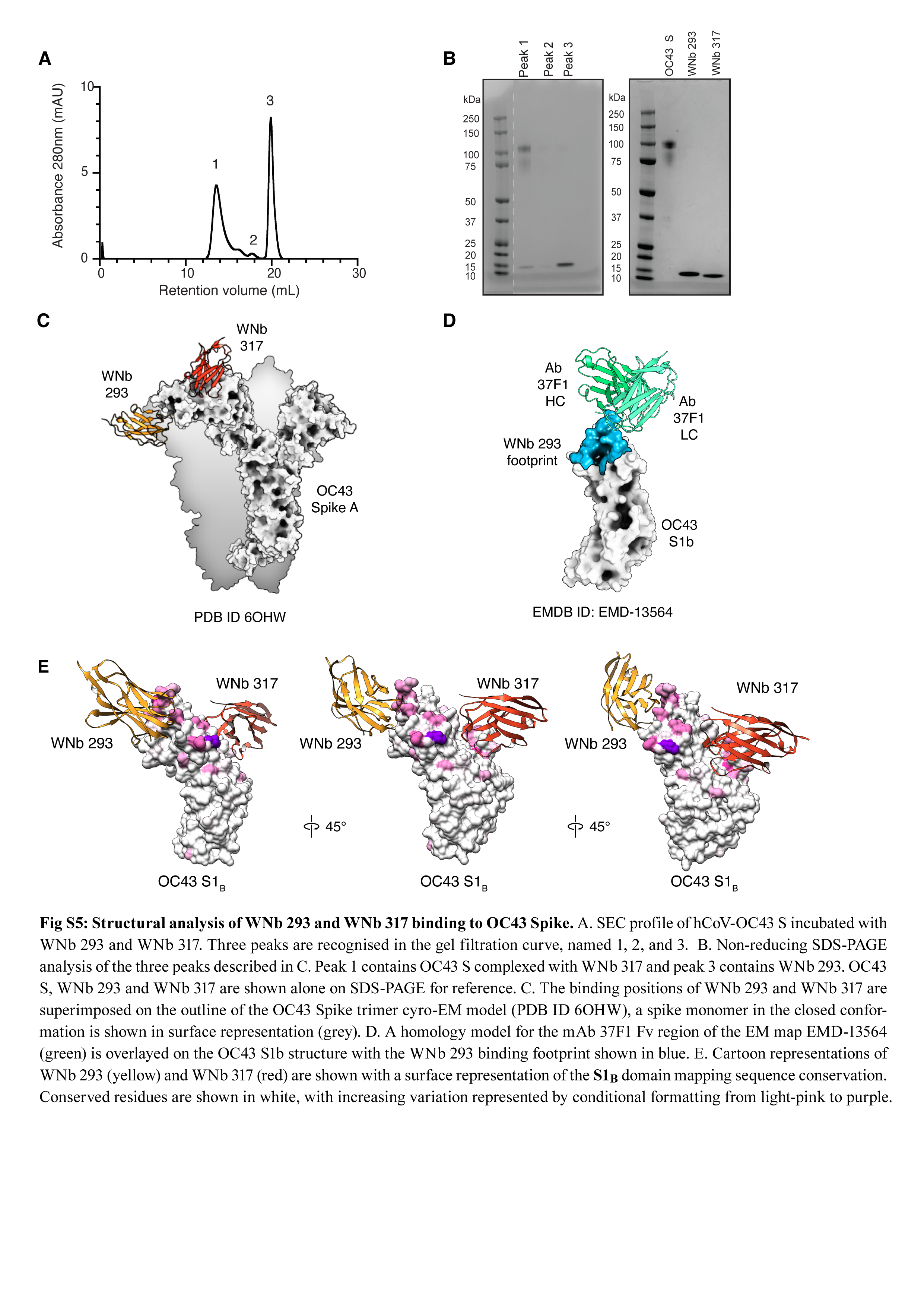

Supplement: Figure S5 — Structural analysis of WNb 293 and WNb 317 binding to OC43 Spike. [file jvi.00531-24-s0005.tif]
