# Supplementary material for: Human coronavirus OC43 nanobody neutralizes virus and protects mice from infection
Source: J Virol. 2024 May 6;98(6):e00531-24. doi: 10.1128/jvi.00531-24 (PMC11237593; doi:10.1128/jvi.00531-24)
Supplement: Table S1 — Summary of interactions between OC43 RBD and nanobodies WNb 293 and WNb 317. [file jvi.00531-24-s0006.docx]

**Supplementary Table 1 | Summary of interactions between OC43 RBD and nanobodies WNb 293 and WNb 317.**

OC43 RBD and WNb 293 nanobody based on the crystal structure PDB 8TZU

| OC43 RBD | Group | WNb 293 | Group | Distance (Å) | |
| --- | --- | --- | --- | --- | --- |
|  |  |  |  |  |  |
| Hydrogen bonds | | | | |  |
| Ser 506 | O | Thr 58 | N | 2.9 | |
| Ser 506 | O | Thr 58 | OG1 | 3.4 | |
| Val 508 | N | Gly 56 | O | 2.7 | |
| Thr 525 | N | Glu 108 | OE2 | 3.8 | |
| Asn 526 | O | Ser 104 | OG | 3.8 | |
| Asn 526 | OD1 | Glu 108 | N | 3.0 | |
| Asn 526 | ND2 | Glu 108 | O | 2.6 | |
| Asn 526 | ND2 | Ser 104 | O | 3.6 | |
| Tyr 527 | N | Tyr 106 | O | 2.9 | |
| Leu 528 | O | Tyr 59 | OH | 3.8 | |
| Thr 529 | N | Gly 103 | O | 3.1 | |
| Cys 530 | N | Thr 57 | OG1 | 2.8 | |
| Asp 531 | OD2 | Ser 52 | OG | 2.6 | |
| Asp 531 | OD2 | Trp 53 | N | 3.6 | |
| Asp 531 | O | Trp 53 | NE1 | 2.8 | |
| Asp 531 | OD2 | Ser 54 | N | 3.1 | |
| Asp 531 | OD2 | Ser 54 | OG | 2.7 | |
| Asp 531 | OD1 | Thr 57 | OG1 | 3.1 | |
| Asp 531 | N | Thr 57 | OG1 | 3.6 | |

| Other OC43 RBD interfacing residues (WNb 293) | | | | | |
| --- | --- | --- | --- | --- | --- |
|  |  |  |  |  |  |
| Gly 55 | Tyr 60 | Trp 100 | Asp 101 | Gly 102 | Trp 105 |
| Pro 107 | Gly 109 |  |  |  |  |

| Other WNb 293 interfacing residues (OC43 RBD) | | | | | |
| --- | --- | --- | --- | --- | --- |
|  |  |  |  |  |  |
| Asn 497 | Phe 498 | Cys 499 | Leu 503 | Gly 505 | Cys 507 |
| Gly 524 | Asn 532 | Leu 533 | Lys 546 | Pro 548 |  |

OC43 RBD and WNb 317 nanobody based on the crystal structure PDB 8TZU

| OC43 RBD | Group | WNb 317 | Group | Distance (Å) |
| --- | --- | --- | --- | --- |
|  |  |  |  |  |
| Hydrogen bonds | | | | |
| Ser 470 | O | Asn 104 | ND2 | 2.9 |
| Val 471 | O | Asn 104 | ND2 | 2.8 |
| Lys 473 | NZ | Asn 104 | O | 2.8 |
| Lys 473 | NZ | Asn 104 | OD1 | 3.8 |
| Lys 473 | NZ | Phe 105 | O | 2.7 |
| Arg 475 | NH1 | Tyr 60 | O | 3.4 |
| Arg 475 | NH2 | Tyr 60 | O | 3.3 |
| Pro 476 | O | Phe 47 | N | 3.6 |
| Ala 477 | O | Arg 110 | NH2 | 3.9 |
| Tyr 487 | OH | Asp 56 | OD1 | 2.6 |
| Gln 489 | OE1 | Trp 53 | NE1 | 3.2 |
| Gln 489 | NE2 | Ser 54 | OG | 3.0 |
| Asp 537 | OD2 | Tyr 100 | OH | 3.7 |
| Thr 540 | OG1 | Tyr 100 | OH | 2.6 |
| Tyr 569 | OH | Asp 108 | OD2 | 3.8 |
| Gln 580 | NE2 | Asp 108 | OD2 | 2.9 |
| Gly 584 | N | Leu 101 | O | 2.9 |
| Trp 585 | N | Leu 101 | O | 3.6 |
| Ser 586 | OG | Tyr 59 | OH | 3.6 |
| Ser 586 | OG | Asp 56 | OD2 | 3.4 |
| Ala 587 | O | Tyr 59 | OH | 2.6 |
| Salt bridges | | | | |
| Arg 413 | NH1 | Asp 56 | OD1 | 3.1 |

| Other OC43 RBD interfacing residues (WNb 317) | | | | | |
| --- | --- | --- | --- | --- | --- |
|  |  |  |  |  |  |
| Thr 28 | Asn 31 | Phe 37 | Glu 44 | Arg 45 | Glu 46 |
| Ala 50 | Ser 52 | Gly 55 | Gly 57 | Ala 61 | Asp 62 |
| Ser 102 | Leu 103 | Pro 106 | Leu 109 |  |  |

| Other WNb 317 interfacing residues (OC43 RBD) | | | | | |
| --- | --- | --- | --- | --- | --- |
|  |  |  |  |  |  |
| Tyr 424 | Gly 478 | Val 479 | Leu 480 | Asp 484 | His 490 |
| Pro 536 | Ile 539 | Lys 566 | Pro 579 | Phe 582 | Leu 583 |
| Asp 588 | Ser 589 |  |  |  |  |
